# Associations Between *FTO* (Fat Mass- and Obesity-Associated) Gene Allelic Load and Heart Rate Responses to Submaximal Exercise in Humans: A Cross-Sectional Study

**DOI:** 10.3390/biology15181607

**Published:** 2026-09-11

**Authors:** Habib Al Ashkar, Nora Kovacs, Karoly Nagy, Roza Adany, Peter Piko

**Affiliations:** 1 Doctoral School of Health Sciences, University of Debrecen, 4032 Debrecen, Hungary; habib.al.ashkar@med.unideb.hu; 2 HUN-REN-UD Public Health Research Group, Department of Public Health and Epidemiology, Faculty of Medicine, University of Debrecen, 4032 Debrecen, Hungaryadany.roza@med.unideb.hu (R.A.); 3 Department of Public Health and Epidemiology, Faculty of Medicine, University of Debrecen, 4032 Debrecen, Hungary; nagy.karoly@med.unideb.hu; 4 National Laboratory for Health Security, Center for Epidemiology and Surveillance, Semmelweis University, 1089 Budapest, Hungary; 5 Department of Preventive Medicine and Public Health, Semmelweis University, 1089 Budapest, Hungary

**Keywords:** heart rate, *FTO* gene, polymorphism, physical activity, exercise response, cardiovascular physiology

## Abstract

Obesity is a widespread condition strongly linked to increased cardiovascular risk, and heart rate (HR) responses to physical activity are widely used physiological indicators of this relationship. Although the role of fat mass- and obesity-associated (*FTO*) gene variants in obesity risk is well established, their potential associations with HR changes during and after physical activity (independent of adiposity) have not yet been fully elucidated. This study examined whether common *FTO* polymorphisms (rs9939609, rs1121980, rs1558902, and rs9941349) and an exploratory *FTO* multi-variant score (MVS*_FTO_*), representing regional variation across the high-LD *FTO* locus, are associated with HR responses to a brief submaximal exercise stimulus. A population-based cohort of Hungarian adults (*n* = 660) completed the YMCA 3-min step test, with HR measured at rest, at first-minute post-exercise (HR_aft_), and at 5- (HR_5min_) and 10-min recovery (HR_10min_). Acute HR response (ΔHR) and percent of predicted maximal HR (HR_max%_) were calculated. Linear regression analyses showed no association between any *FTO* variant and resting HR (all *p* > 0.05). In contrast, all four candidate SNPs demonstrated significant negative associations with HR_aft_ (β ≈ −3.6 to −4.2, all *p* ≤ 0.005), ΔHR (β ≈ −3.7 to −3.9, *p* ≤ 0.005), and HR_max%_ (β ≈ −2.5, *p* = 0.001). The MVS*_FTO_* revealed even more pronounced and consistent associations: higher MVS*_FTO_* burden was associated with lower HR_aft_ (β = −4.19, *p* = 0.001), HR_5min_ (β = −2.06, *p* = 0.004), HR_10min_ (β = −1.15, *p* = 0.009), ΔHR (β = −4.04, *p* = 0.001), and HR_max%_ (β = −2.47, *p* = 0.001). Exploratory four-way mediation decompositions and saturated structural equation models indicated that these inverse associations remained statistically robust after multivariable adjustment for measured BMI and leisure-time physical activity (all direct paths *p* ≤ 0.001). Furthermore, multi-trait latent cluster analysis identified distinct phenotypic profiles consistent with these findings, showing that individuals with high MVS*_FTO_* burden and moderate physical activity exhibit blunted exercise HR reactivity across recovery phases. Rather than establishing biological causality or clinical cardiovascular benefit, these observational findings demonstrate that regional *FTO* locus variation captured by the multi-variant score is statistically associated with inter-individual variability in short-term post-exercise HR reactivity, providing hypothesis-generating insights that warrant validation in prospective cohorts and interventional exercise trials.

## Simple Summary

People differ in how their heart responds to physical exertion. In this study, we explored whether common variations in the *FTO* gene, widely known for its role in body weight regulation, are associated with heart rate responses during and after a standardized submaximal step test in a population-based cohort of 660 adults. While resting heart rate was unaffected by genetic variation, carrying a higher cumulative load of coded *FTO* alleles across candidate loci was statistically associated with lower first-minute post-exercise heart rate, a blunted acute heart rate rise (ΔHR), and a lower percentage of predicted maximal heart rate. Delayed post-exercise recovery parameters exhibited only nominal, exploratory associations. These primary associations remained statistically robust after adjusting for measured body mass index, central adiposity, and leisure-time physical activity, with mediation models indicating only modest indirect contribution through body mass. Furthermore, phenotypic cluster analysis was consistent with these patterns, indicating that individuals with high genetic locus burden and moderate physical activity maintained lower post-exercise heart rate reactivity. Given the cross-sectional nature of this study, these observational findings represent exploratory physiological associations rather than confirmatory evidence of altered cardiorespiratory fitness, faster recovery kinetics, or clinical cardiovascular benefit. They provide a hypothesis-generating foundation for future prospective and interventional studies investigating exercise-induced autonomic regulation.

## 1. Introduction

Globally, obesity and physical inactivity are on the rise, undermining cardiovascular fitness and heavily contributing to the growing global burden of cardiometabolic diseases. According to recent global epidemiological estimates, more than one billion adults live with obesity, and this figure is projected to exceed 1.5 billion by 2035 [1]. This pandemic is driven by complex environmental and lifestyle changes [2], particularly modern dietary transitions characterized [3] by high intake of energy-dense, ultra-processed foods, excess saturated fats, and refined sugars, alongside inadequate consumption of micronutrients and dietary fiber [4]. Concurrently, modern occupational structures, urbanization, and prolonged screen time have led to widespread sedentary behavior, with nearly one-third of the global adult population failing to meet the World Health Organization’s recommended physical activity guidelines [5].

These unhealthy dietary behaviors and physical inactivity do not act in isolation; they interact synergistically with key metabolic comorbidities, including essential hypertension, atherogenic dyslipidemia, insulin resistance, and type 2 diabetes mellitus [6]. Epidemiological studies [7,8] demonstrate that the co-occurrence of obesity and these metabolic disorders significantly accelerates vascular damage, impairs endothelial function, and alters cardiac autonomic tone. Consequently, individuals experience reduced cardiorespiratory fitness, diminished exercise tolerance, and an elevated risk of adverse cardiovascular events, contributing to millions of premature deaths globally each year [1,5].

Heart rate (HR) dynamics during and after physical activity are widely used physiological indicators of cardiovascular health and autonomic balance. HR responses reflect the integrated effects of body composition, habitual physical activity, diet-related metabolic status, and inter-individual biological variability [9,10]. Although these responses are influenced by multiple behavioral and physiological factors, emerging evidence suggests that genetic variation may also contribute to differences in HR patterns during exercise [11].

The fat mass- and obesity-associated (*FTO*) gene was the first locus identified for common polygenic obesity and remains one of the most extensively studied genetic risk factors. Common *FTO* variants (such as rs9939609, rs1121980, and rs1558902) are strongly associated with elevated body mass index (BMI), central adiposity, and overall metabolic risk, with risk-allele carriers exhibiting a 20–30% higher likelihood of developing obesity [12].

Beyond its established role in adiposity, emerging evidence indicates that physical exertion acutely alters tissue-specific *FTO* expression in skeletal muscle and adipose tissue, while experimental models demonstrate distinct links to autonomic regulation and cardiac electrophysiology [13,14,15,16]. These physiological observations provide a strong biological rationale for testing whether common *FTO* genetic variants are associated with short-term heart rate reactivity and post-exercise recovery dynamics during standardized exercise.

Although the metabolic consequences of *FTO* variation are well established, several observational studies suggest that its cardiometabolic relevance may not be mediated exclusively by adiposity [16,17]. While *FTO* risk alleles have been linked to long-term cardiovascular outcomes primarily through higher BMI [18], persistent associations with autonomic and vascular endpoints have been reported even after statistical adjustment for body mass. However, whether *FTO* genetic variation is statistically associated with short-term cardiovascular autonomic reactivity, specifically acute heart rate dynamics during and after standard exercise, remains a key research gap.

To address this gap, the present cross-sectional study evaluated whether common *FTO* gene variants and a cumulative allelic load score are associated with heart rate responses to a brief submaximal exercise stimulus in adults. Four specific single nucleotide polymorphisms (rs9939609, rs1121980, rs1558902, and rs9941349) were selected based on their established strong associations with obesity and cardiometabolic traits in European and Hungarian populations, as well as their representation of a high linkage disequilibrium (LD) region within the *FTO* locus. We tested the hypothesis that individual *FTO* variants are associated with acute cardiovascular reactivity to exercise, and explored whether a high-LD multi-variant score captures cumulative regional variation.

In our analytical hierarchy, individual *FTO* SNPs and the exploratory multi-variant score (MVS*_FTO_*) served as indicators of regional locus burden, while first-minute post-exercise and acute post-exercise heart rate responses were defined as the primary physiological outcomes. Secondary analyses evaluated post-exercise recovery heart rate trajectories at 5 min and 10 min, alongside the percentage of predicted maximum heart rate. Further exploratory analyses assessed statistical gene–environment and gene–body composition interactions, potential mediation paths via body mass index and leisure-time physical activity, structural equation modeling (SEM) network structures, and multi-trait latent cluster profiles. Across all analytical tiers, a central objective was to determine whether these statistical associations remained robust after adjustment for body composition and habitual physical activity, without implying biological independence or causal pathways.

## 2. Materials and Methods

### 2.1. Study Design and Population

This cross-sectional analysis used data from a population-based health survey conducted in 2018 in northeastern Hungary, targeting adults aged 20–64 years from the Hungarian general population and the Roma minority [19]. Data collection followed a standardized three-pillar protocol (questionnaires, physical examination, laboratory testing) previously validated in cardiometabolic research [20,21].

From the initial recruited sample of 832 individuals, 35 declined participation prior to examination. Of the remaining 797 participants, 127 were excluded due to incomplete heart rate measurement values across the four required time points (resting, first-minute post-exercise, 5-min recovery, and 10-min recovery). A further 10 participants were excluded due to missing genotyping data. Consequently, complete case analysis was applied, resulting in a final analytical sample of 660 individuals (82.8% of the 797 individuals who proceeded to examination). A detailed participant-flow diagram is provided in Supplementary Figure S1.

Eligibility criteria included community-dwelling adults aged 20–64 years. Prior to physical testing, participants were screened for physical limitations, and the test was carried out under the supervision of a general practitioner. Rather than using standardized pre-exercise screening questionnaires, clinical conditions were assessed by self-report, and baseline resting blood pressure was measured in all participants prior to exertion. Individuals deemed at potential health risk or unable to perform or complete the YMCA 3-min step test due to pre-existing physical disabilities, acute musculoskeletal problems, or severe self-reported cardiovascular or medical contraindications did not undergo the exercise protocol. The comprehensive inclusion of anthropometric (BMI, waist-to-height ratio), hemodynamic (systolic and diastolic blood pressure), and lifestyle covariates ensured robust adjustment for underlying cardiometabolic burden within the available data.

### 2.2. Survey Design and Data Collection Protocols

Procedures adhered to EHIS/EHES standards [19,22]. Data were collected through questionnaires, physical examinations, and laboratory testing. Anthropometric and blood pressure measurements were obtained using standardized protocols, and BMI was calculated. Participants completed validated questionnaires on lifestyle and psychological well-being, including the 12-item General Health Questionnaire (GHQ-12) [23] and International Physical Activity Questionnaire—Long Form (IPAQ-LF) [24]. Fasting blood samples were analyzed for cardiometabolic biomarkers, and genomic DNA was extracted for genotyping. A detailed description of the questionnaire structure and measurement protocols can be found in the methodological summary published by Ádány et al. [19].

### 2.3. SNP Selection and Genotyping

Four *FTO*-linked SNPs (rs9939609, rs1121980, rs1558902, and rs9941349) were selected based on prior studies in Hungarian and Roma populations [25,26] and supporting GWAS evidence [27].

Genotyping was performed on a MassARRAY platform (Sequenom, Inc., San Diego, CA, USA) using iPLEX Gold chemistry by the Mutation Analysis Facility (MAF) service provider at the Karolinska Institute (Solna, Sweden). The Facility conducted validation, concordance analysis, and quality control according to their established standard protocols. Among the 660 participants included in the complete-case analysis, genotype completeness was 100%; 10 individuals with missing genotyping data had been excluded during initial cohort filtering.

For each polymorphism, the genetic variation was parameterized as the number of coded alleles under an additive linear model (0, 1, or 2 coded alleles). The coded allele frequencies ranged between 42% and 44% across the cohort (rs1558902-A: 43%; rs1121980-A: 44%; rs9939609-A: 42%; and rs9941349-T: 42%), and observed genotype distributions showed excellent concordance with Hardy–Weinberg Equilibrium expectations across all loci (all *p* ≥ 0.470; Supplementary Table S1).

Pairwise Linkage Disequilibrium (LD) was evaluated using D’, D, and Pearson’s correlation coefficient (r). Pairwise LD correlation statistics were extremely strong across all locus pairs (r = 0.948–0.966; D’ = 0.953–0.997; all *p* < 0.001), confirming a single high-LD genomic block (Supplementary Table S1). We explicitly define “r” as Pearson’s correlation coefficient between allele dosages rather than r^2^ (coefficient of determination). For more details see Supplementary Table S2.

In single-locus regression models, all four SNPs consistently exhibited negative associations with post-exercise heart rate reactivity. However, when all four SNPs were entered simultaneously into a fully adjusted multi-SNP model, severe collinearity (Variance Inflation Factor (VIF) = 15.72) led to parameter instability and statistical suppression for rs9941349 (Supplementary Table S1). This confirmed that individual SNPs convey largely redundant information, strongly justifying the construction of a cumulative multi-variant score (MVS*_FTO_*, range 0–6 coded alleles) as an exploratory summary metric of overall locus burden rather than relying on simultaneous multi-locus regression.

### 2.4. Heart Rate Assessment

Cardiovascular reactivity and autonomic dynamics in response to physical exertion were evaluated using the standardized YMCA 3-min step test protocol [28,29]. All field assessors (medical research personnel) underwent standardized pre-study training to ensure high inter-rater reliability in cadence enforcement and manual radial pulse palpation. Each testing session commenced with a 2-min seated rest period on a chair, after which baseline resting heart rate (HR_rest_) was manually measured in the seated position via radial pulse palpation for 60 s. Participants were then instructed to step up and down a 30 cm box a total of 72 times over a 3-min duration. The stepping rate was calibrated to 24 steps per minute using an auditory metronome set at 96 beats per minute (4 metronome clicks representing one complete stepping cycle). Continuous supervision was provided to ensure strict adherence to the prescribed workload and cadence.

Immediately upon test completion, participants sat down, remained motionless for 5 s, and first-minute post-exercise heart rate (HR_aft_) was manually recorded via radial pulse palpation for exactly 60 s. Post-exercise pulse measurements were repeated at 5 min (HR_5min_) and 10 min (HR_10min_) post-exercise using identical 60-s radial palpation intervals while participants remained seated.

Acute heart rate response (ΔHR) was defined as the net heart rate increase above baseline (ΔHR = HR_aft_ − HR_rest_). Net heart rate elevations at 5- and 10-min post-exercise were calculated relative to resting baseline (ΔHR_5min_ = HR_5min_ − HR_rest_ and ΔHR_10min_ = HR_10min_ − HR_rest_). These metrics represent residual cardiovascular elevation relative to baseline resting tone rather than conventional post-exercise recovery slopes. Predicted maximum heart rate (HR_max_) [30] and percentage of predicted maximum heart rate (HR_max%_) [31] were calculated using age-adjusted physiological formulas:

$${\mathrm{HR}}_{\max}=220-\mathrm{age}\;(\mathrm{years})$$

$${\mathrm{HR}}_{\max\%}=\frac{{\mathrm{HR}}_{\mathrm{aft}}}{{\mathrm{HR}}_{\max}}\times 100$$

Participants were initially categorized into seven YMCA fitness levels (very poor, poor, below average, average, above average, good, and excellent) based on age- and sex-specific post-exercise heart rate cut-offs derived from the YMCA fitness guidelines [28,29]. For analytical and statistical testing purposes, these seven original categories were subsequently grouped into three strata: very poor/poor (*n* = 191), below average/average/above average (*n* = 295), and good/excellent (*n* = 174). The detailed normative post-exercise heart rate cut-offs (in beats per minute) utilized for this classification across different age groups for women and men are provided in Supplementary Table S3.

This classification is based on standardized normative values, with post-exercise heart rate evaluated against 10-year age intervals with separate criteria for men and women. The stratification enabled standardized comparisons using sex- and age-specific YMCA norms, allowing participants’ acute exercise responses to be evaluated against public health benchmarks and facilitating subgroup trend analyses across functional fitness tiers.

### 2.5. Multi-Variant Score Construction

To evaluate cumulative regional variation across the *FTO* locus, an exploratory multi-variant score (MVS*_FTO_*) was constructed from the genotyped candidate variants. Genotypes were parameterized assuming an additive linear allele-dosage model (0, 1, or 2 coded alleles).

The rationale for allele selection stemmed from single-SNP versus multi-SNP modeling behavior. When evaluated individually in single-locus models, the obesity-risk rs9941349-T allele exhibited a negative association with acute heart rate reactivity (ΔHR; β = −3.111, *p* = 0.020), whereas its complementary rs9941349-C allele was positively associated. However, when all four candidate loci were entered simultaneously into a joint multi-SNP model, extreme linkage disequilibrium (r = 0.948–0.966) and severe collinearity (VIF = 15.72) caused statistical suppression: the coefficient of rs9941349-T shifted to positive (β = 9.883, *p* = 0.074), while the effect of rs9941349-C correspondingly became negative in the presence of the other SNPs (Supplementary Table S1).

Consequently, to ensure directional alignment with the negative effects of rs1121980-A and rs1558902-A in a multi-locus context, stepwise forward variable selection incorporated the rs9941349-C allele into the composite score. Candidate variants were iteratively evaluated and retained based on incremental variance explained (ΔR^2^), improvement in Adjusted R^2^, and reduction in Root Mean Squared Error (RMSE) beyond baseline sociodemographic, lifestyle, and resting hemodynamic covariates (R^2^ = 0.0868).

The finalized score represents an unweighted exploratory summary metric of coded alleles across retained loci (theoretical range: 0–6) derived internally from incremental multi-locus model selection. Because this composite index was optimized internally within the present dataset, it serves strictly as an exploratory regional burden index rather than an externally validated score, requiring future independent replication. Participants were categorized into Low (1–2 alleles), Intermediate (3 alleles), and High (4–6 alleles) tiers for subgroup and trend evaluations.

### 2.6. Statistical Analysis

Descriptive statistics are presented as mean ± standard deviation (SD) for continuous variables and as frequencies with percentages for categorical variables. Categorical variables were compared using chi-squared (χ^2^) tests. Data distribution and normality were evaluated using Kolmogorov–Smirnov tests alongside visual inspection of Q-Q plots and skewness/kurtosis diagnostics. For non-normally distributed continuous variables, two-group comparisons were evaluated using the Mann–Whitney U test, omnibus differences across three or more categories were evaluated using the Kruskal–Wallis test, and progressive ordered trends across categories were formally assessed using the non-parametric Jonckheere–Terpstra trend test [32].

To satisfy normality and homoscedasticity assumptions required for multivariable linear regression modeling, non-normally distributed continuous variables were normalized using Templeton’s two-step transformation method [33]. Briefly, this non-parametric transformation first converts raw continuous values into fractional/percentile ranks to obtain uniformly distributed probabilities (Step 1), and subsequently applies the inverse-normal cumulative distribution function to yield standardized, normally distributed Z-scores (Step 2). To preserve direct clinical interpretability, the resulting normal scores were linearly rescaled back using the original mean and standard deviation of each variable. Consequently, transformed continuous variables (including sociodemographic/clinical covariates and all exercise heart rate outcomes) retained their original metric scale, and multivariable linear regression estimates (β-coefficients and 95% CIs) are reported in their original physical units (e.g., beats per minute for heart rate traits).

Associations between *FTO* variants and post-exercise heart rate outcomes were examined using fully adjusted multivariable linear regression models (*n* = 660). Models controlled for sociodemographic and lifestyle confounders, including self-reported ethnicity (Hungarian vs. Roma), sex, age, educational attainment (3 categories), psychological distress (GHQ-12 Likert scale), smoking status, leisure-time physical activity, and domestic physical activity. Rest hemodynamic parameters (systolic and diastolic blood pressure) and adiposity indices (BMI and waist-to-height ratio [WHtR]) were included as controlled baseline clinical covariates. Post-estimation regression diagnostics confirmed model adequacy, including the normality and homoscedasticity of residuals, and confirmed the absence of severe multicollinearity (baseline model mean VIF = 1.94, all individual covariate VIFs < 3.88). To confirm that results were not driven by population stratification, sensitivity analyses were performed by fitting fully adjusted regression models separately within the Hungarian general population and the Roma minority.

Sequential adjustment models were implemented to evaluate potential overadjustment across demographic, hemodynamic, and lifestyle covariates (Supplementary Table S4). Exploratory mediation pathways were evaluated using the regression-based four-way decomposition approach (Stata v17.0, StataCorp LLC, College Station, TX, USA; med4way procedure using the Delta method) with BMI and LTPA as potential mediators, decomposing total effects into controlled direct effects (CDE), reference and mediated interactions (INTref, INTmed), and pure indirect effects (PIE). Potential moderation effects (MVS*_FTO_* × BMI, MVS*_FTO_* × LTPA, and three-way interaction terms) were tested using multivariable factorial regression models. Subsequently, structural equation modeling was performed using maximum likelihood estimation to simultaneously examine multivariate network pathways. Given the fully saturated structure of the structural equation models (df = 0), SEM was applied strictly as a path-analytic parameter estimation framework across the observed system rather than for confirmatory goodness-of-fit evaluation.

To evaluate hypotheses rigorously while controlling for Type I error inflation across multiple loci, cardiovascular outcomes, and complex model extensions, statistical testing was hierarchically structured into pre-specified analytical families. Within this framework, multivariable linear regressions evaluating individual genotyped *FTO* variants against primary acute exercise reactivity outcomes (HR_aft_, ΔHR, and HR_max%_) served as the primary hypothesis tests, adjusted for multiple comparisons using a strict Bonferroni-corrected threshold of *p* < 0.0125 (alpha = 0.05/4 candidate loci). Secondary single-SNP analyses evaluating baseline resting tone (HR_rest_) and post-exercise recovery heart rate trajectories (HR_5min_, HR_10min_, ΔHR_5min_, and ΔHR_10min_) were controlled using the Benjamini–Hochberg False Discovery Rate (FDR) procedure (q < 0.05). Because the MVS*_FTO_* was internally derived based on incremental performance for ΔHR within this cohort, all MVS*_FTO_* analyses were designated a priori as exploratory and hypothesis-generating rather than confirmatory, requiring independent validation. To maintain a structured evaluation across endpoints, exploratory MVS*_FTO_* models were assessed within this identical two-tiered outcome-family framework (family-wise threshold *p* < 0.0125 for primary reactivity traits, and Benjamini–Hochberg FDR q < 0.05 for secondary recovery traits).

All subgroup interaction models, adiposity mediation pathways (BMI and WHtR), and directional structural equation modeling paths were designated a priori as exploratory and hypothesis-generating; nominal *p*-values, 95% confidence intervals, and non-parametric bootstrap standard errors (1000 replications for SEM paths) are reported.

Graphical visualizations and statistical plots were generated using GraphPad Prism v10.1 (GraphPad Software, San Diego, CA, USA).

### 2.7. Use of Generative AI

During the preparation of this manuscript, Microsoft Copilot (version: June 2026) was utilized to assist with text refinement and language editing. All AI-generated content was reviewed and edited thoroughly by the authors. The authors affirm full responsibility for the accuracy, integrity, and originality of the final manuscript.

## 3. Results

### 3.1. Characteristics of Sample Population

The sociodemographic, physical, and behavioral characteristics of the final analytical cohort across YMCA post-exercise heart rate fitness categories—Very poor/Poor (*n* = 191), Above average/Average/Below average (*n* = 295), and Good/Excellent (*n* = 174)—are presented in Table 1.

The proportion of Roma participants showed no significant linear trend across fitness tiers (ranging between 39.66% and 59.77%, *p*-trend = 0.999). In contrast, women were significantly more prevalent in the Good/Excellent group (79.06%) compared to the Average (56.27%) and Very poor/Poor (67.24%) groups (*p*-trend = 0.010). Educational attainment did not differ systematically across the categories (*p*-trend = 0.708), with the majority of participants reporting primary or lower education (45.76% to 60.92%). Participants in higher fitness categories were slightly older, with median age increasing progressively from 41.50 years (IQR: 38.00–44.00) in the Poor tier to 46.00 years (IQR: 45.00–50.00) in the Good/Excellent tier (*p*-trend = 0.009).

While height, weight, and body mass index showed no significant difference across categories, waist circumference was markedly lower in the Good/Excellent fitness group (median 90.00, IQR: 89.00–94.00) relative to the Poor group (median 97.00, IQR: 93.00–100.00; *p*-trend < 0.001). Resting systolic blood pressure remained comparable across categories (*p*-trend = 0.759), whereas diastolic blood pressure showed a modest inverse trend across fitness tiers (*p*-trend = 0.012).

Both physical activity domains demonstrated strong, positive trends across fitness tiers: median domestic physical activity rose from 1650.00 MET-min/week (IQR: 1440.00–1890.00) in the Poor group to 2510.00 MET-min/week (IQR: 2070.00–2940.00) in the Good/Excellent group (*p*-trend < 0.001), and leisure-time physical activity similarly increased from 231.00 to 495.00 MET-min/week (*p*-trend < 0.001). Psychological distress scores (GHQ-12) did not vary significantly among the fitness groups (median 9.50 to 10.00, *p*-trend = 0.272). For more details, see Table 1.

### 3.2. Associations Between FTO SNPs and Heart Rate Traits

**Figure 1:**
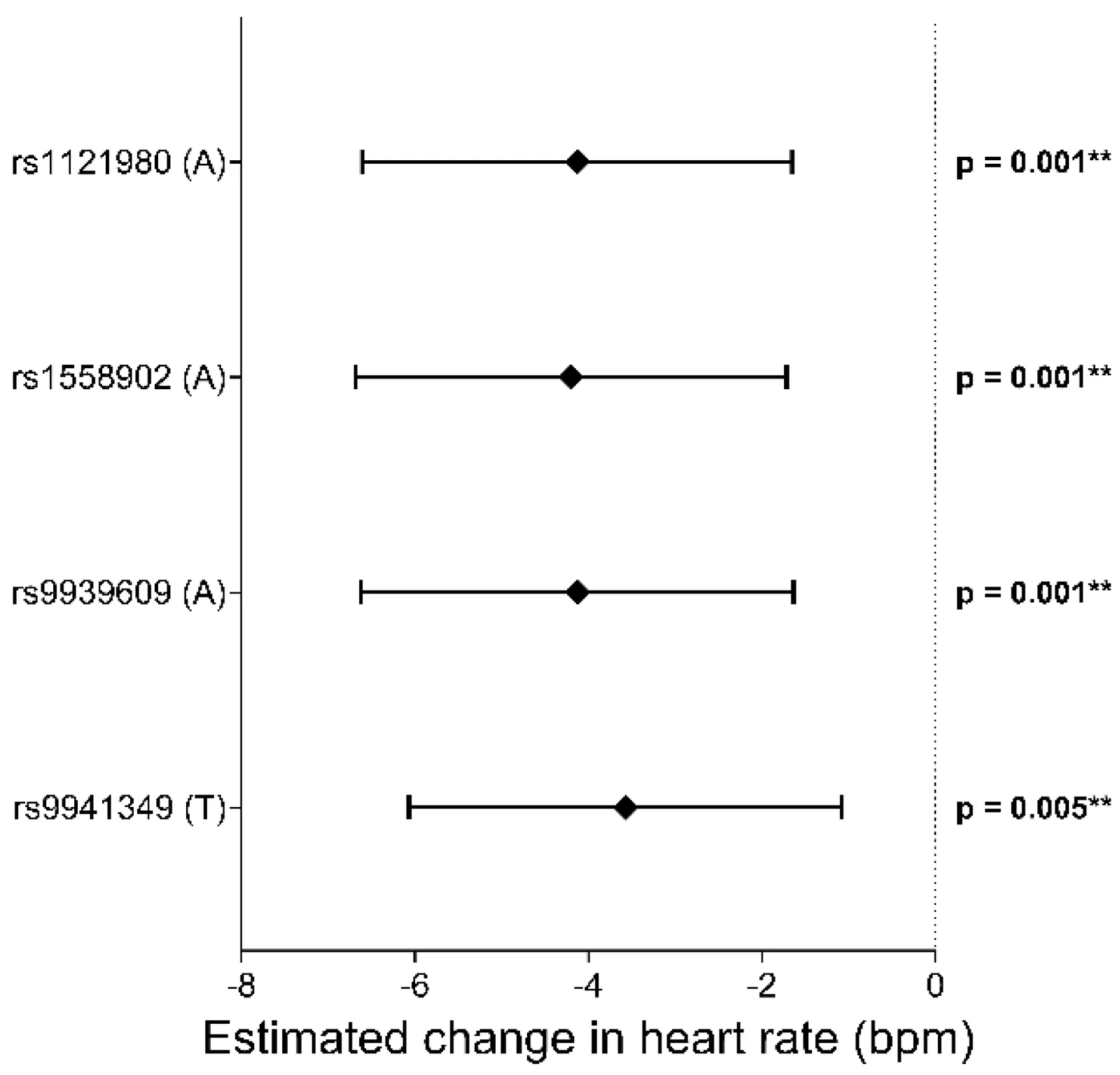
Association of *FTO* SNPs with first-minute post-exercise heart rate. The figure shows the β-values (in beats per minute) and the 95% confidence intervals obtained from the linear regression analyses. **: Bonferroni-corrected significance (*p* < 0.0125).

**Figure 2:**
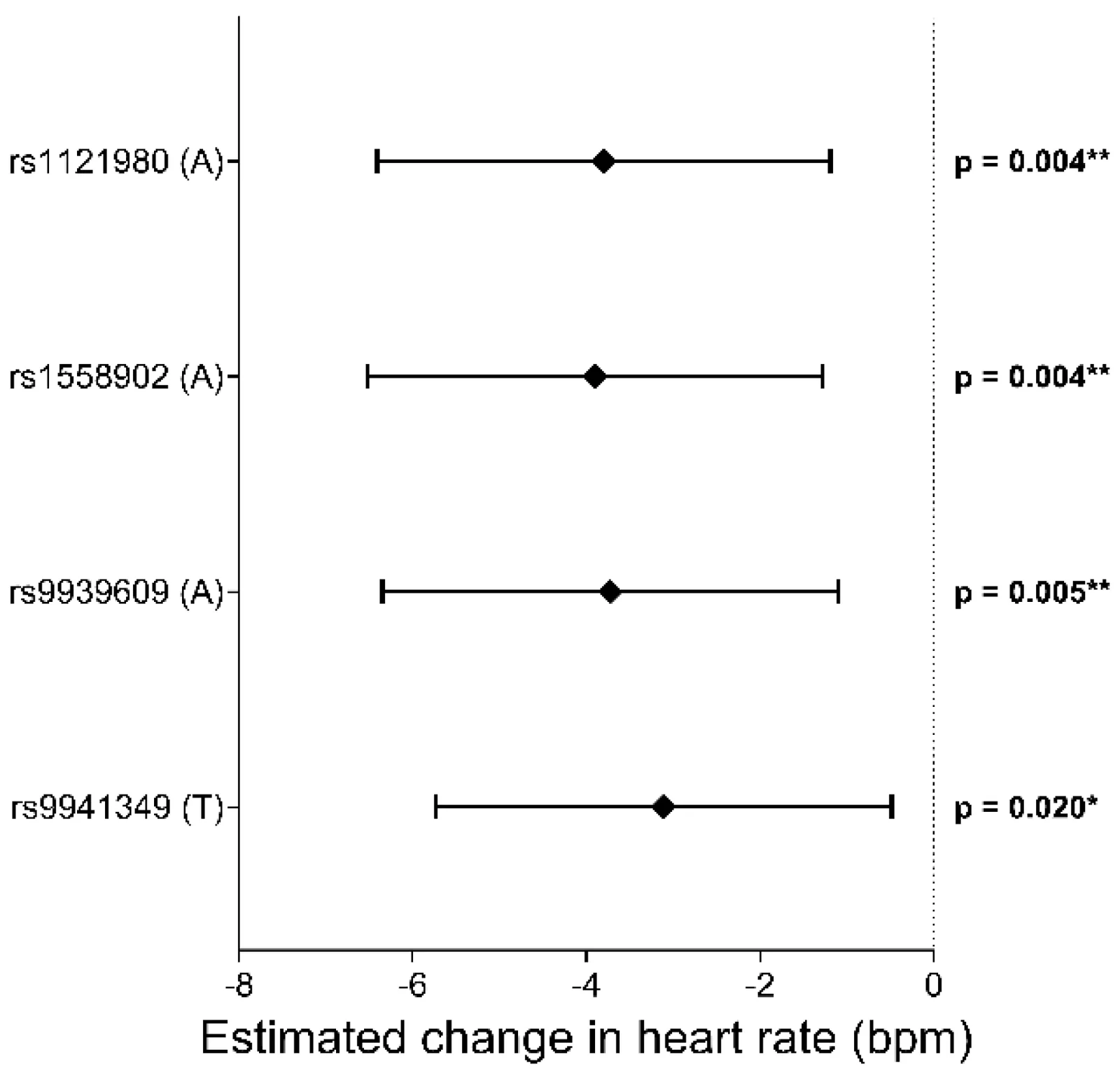
Association of *FTO* SNPs with delta heart rate (ΔHR). The figure shows the β-values (in beats per minute) and the 95% confidence intervals obtained from the linear regression analyses. *: nominally significant (*p <* 0.05); **: Bonferroni-corrected significance (*p <* 0.0125).

**Figure 3:**
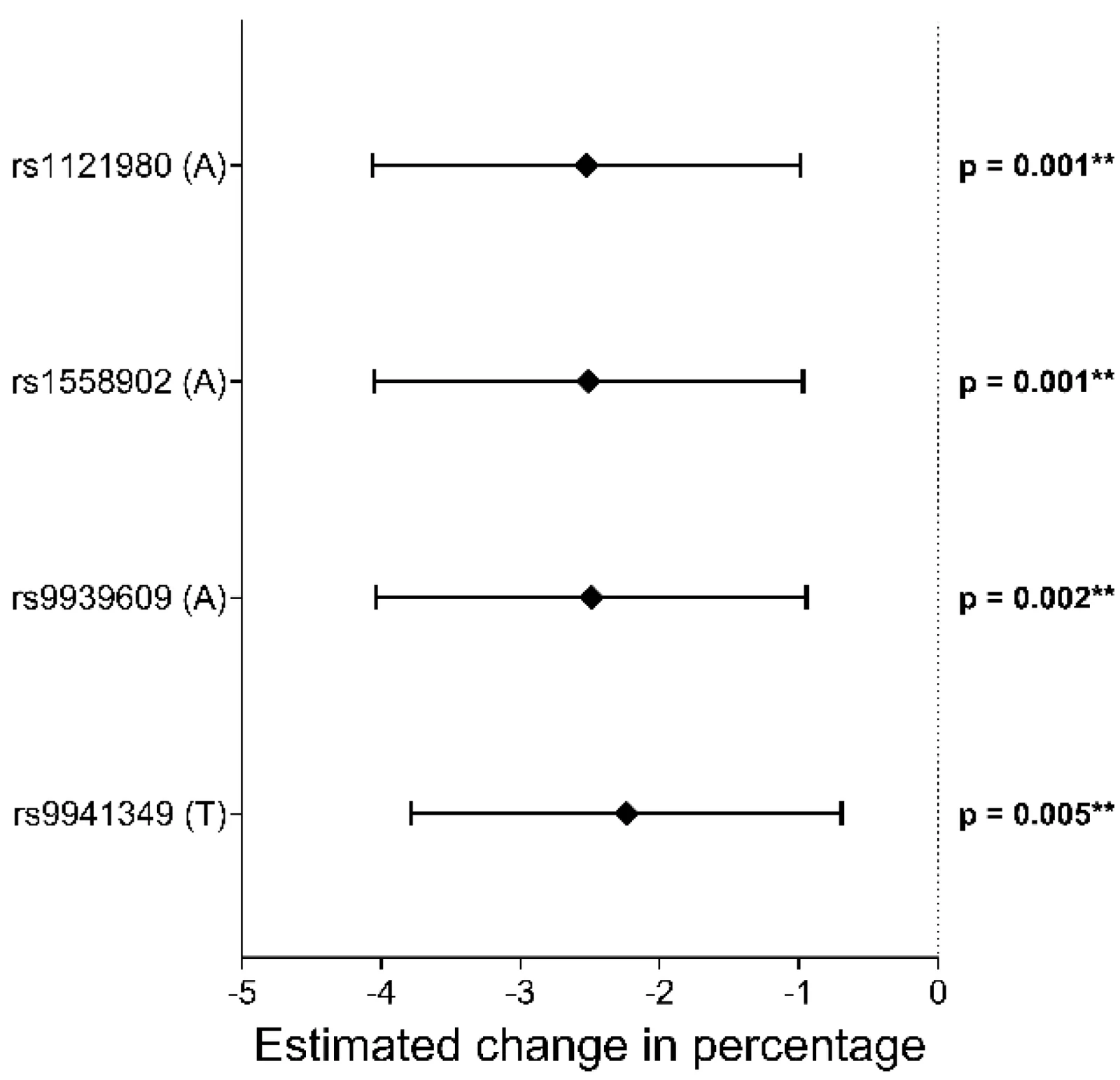
Association of *FTO* SNPs with percent of predicted maximum heart rate. The figure shows the β-values (in percentage points) and the 95% confidence intervals obtained from the linear regression analyses. **: Bonferroni-corrected significance (*p <* 0.0125).

Fully adjusted multivariable linear regression analyses revealed robust negative associations between all four candidate *FTO* SNPs and the primary post-exercise reactivity parameters. Specifically, HR_aft_ was significantly associated with rs1121980 (β = −4.129, *p* = 0.001), rs1558902 (β = −4.199, *p* = 0.001), rs9939609 (β = −4.127, *p* = 0.001), and rs9941349 (β = −3.571, *p* = 0.005), with all associations remaining significant after strict Bonferroni correction (*p* < 0.0125; Figure 1). Similarly, inverse associations with ΔHR remained significant after Bonferroni correction for rs1121980 (β = −3.797, *p* = 0.004), rs1558902 (β = −3.882, *p* = 0.004), and rs9939609 (β = −3.712, *p* = 0.005), while rs9941349 was nominally significant but did not withstand Bonferroni correction (β = −3.119, *p* = 0.020; Figure 2). For HR_max%_, all four loci remained statistically significant after Bonferroni correction (all *p* ≤ 0.005; Figure 3).

In secondary analyses evaluating baseline tone and post-exercise recovery kinetics, multivariable linear regression models showed no association between any candidate *FTO* variant and baseline resting heart rate (all *p* > 0.05, q > 0.05; Supplementary Figure S2). For the recovery phase, associations were evaluated under the Benjamini–Hochberg FDR procedure (q < 0.05). At 5 min of recovery, significant inverse associations were observed across candidate loci (rs9939609: β = −1.961, *p* = 0.010, q = 0.022; rs1558902: β = −1.946, *p* = 0.011, q = 0.022; rs1121980: β = −1.894, *p* = 0.013, q = 0.022; and rs9941349: β = −1.503, *p* = 0.049, q = 0.049), all meeting the FDR significance threshold (Supplementary Figure S3). At 10 min of recovery, inverse associations remained significant under FDR for rs1558902 (β = −1.102, *p* = 0.018, q = 0.029), rs9939609 (β = −1.085, *p* = 0.019, q = 0.029), and rs1121980 (β = −1.061, *p* = 0.022, q = 0.029), while rs9941349 showed a non-significant trend (β = −0.854, *p* = 0.063, q = 0.063; Supplementary Figure S4). Regarding residual elevation above rest, ΔHR_5min_ showed a nominal association with rs1558902 (β = −1.650, *p* = 0.048, q = 0.081; Supplementary Figure S5) and non-significant trends for the remaining loci (*p* = 0.056–0.193, q ≥ 0.081), whereas ΔHR_10min_ was not significantly associated with any locus (*p* = 0.062–0.114, q ≥ 0.095; Supplementary Figure S6). Consequently, these secondary recovery dynamics represent modest exploratory trends.

In sequential adjustment analyses, the associations of rs1121980, rs1558902, rs9939609, rs9941349, and the MVS*_FTO_* with HR_aft_ remained directionally consistent and statistically significant across all models, including the fully adjusted model (Supplementary Table S4).

### 3.3. FTO Multi-Variant Score Construction

Given the very high LD among the four *FTO* variants, the MVS*_FTO_* was designed to capture cumulative allelic load within a single high-LD region rather than independent genetic effects. Variant inclusion was guided by regional LD structure, directional alignment within multi-locus modeling, and incremental model performance on ΔHR.

Stepwise selection started with the lead reference variant rs1121980-A (R^2^ = 0.0984, Adj. R^2^ = 0.081, ΔR^2^ = 0.0116 over baseline, RMSE = 23.902, *p* = 0.0044). Addition of rs1558902-A incrementally improved model fit (R^2^ = 0.0989, Adj. R^2^ = 0.082, RMSE = 23.895, *p* = 0.0037). Adding rs9941349-C achieved the highest overall explanatory power (R^2^ = 0.1016, Adj. R^2^ = 0.085, cumulative ΔR^2^ = 0.0149 over baseline, RMSE = 23.858, *p* = 0.0012), leading to the retention of these three variants in the finalized MVS*_FTO_*. Conversely, rs9939609 was excluded because its inclusion reduced model performance (R^2^ dropped to 0.0987, RMSE increased to 23.898) due to extreme collinearity with reference loci. Detailed model statistics are presented in Table 2.

In stratified sensitivity analyses, the inverse association between MVS*_FTO_* and ΔHR was directionally consistent across both the Hungarian general population (β = −2.65, 95% CI: −5.31–0.02, *p* = 0.051) and Roma minority (β = −3.94, 95% CI: −7.99–0.11, *p* = 0.056) sample groups.

The sociodemographic, physical, and behavioral characteristics of the study population were examined across MVS*_FTO_* groups. Participants were stratified into three groups based on the score value of MVS*_FTO_*: low: 1–2 (*n* = 218), intermediate: 3 (*n* = 305), and high: 4–6 (*n* = 137).

Roma representation was stable across groups (47.87–54.01%, *p* = 0.898). Women were slightly more common in the high-MVS*_FTO_* group (67.43%) than in the intermediate- (65.90%) or low-MVS*_FTO_* (62.77%) groups, but not significantly (*p* = 0.388). Educational attainment was evenly distributed, with no trend detected (*p* = 0.271).

Age was comparable (42.03–43.55 years, *p* = 0.294). BMI increased across MVS*_FTO_* categories, peaking in the high-MVS*_FTO_* group (28.75 kg/m^2^; *p* = 0.015), while waist-to-height ratio and blood pressure showed no differences.

Domestic activity was similar across groups, but LTPA rose significantly, highest in the high-MVS*_FTO_* group (1511.12 MET-min/week; *p* = 0.028). Psychological well-being (GHQ-12) did not differ (*p* = 0.883). For more details see Supplementary Table S5.

HR parameters varied significantly across MVS*_FTO_* groups. HR_rest_ remained stable across groups (~77 bpm), but HR_aft_ declined progressively with increasing MVS*_FTO_* (115.16 bpm in low-MVS*_FTO_* vs. 104.10 bpm in high-MVS*_FTO_*; *p* < 0.001). Similar downward trends were observed at 5- and 10-min of post-exercise (*p* = 0.003 and *p* = 0.022, respectively). ΔHR was most pronounced in the low-MVS*_FTO_* group (37.48 bpm) and least in the high-MVS*_FTO_* group (26.71 bpm; *p* = 0.001). Percent of predicted maximum heart rate similarly decreased from 65.37% to 58.71% across tiers (*p* < 0.001). For more details see Table 3.

Associations between the MVS*_FTO_* and HR parameters are presented in Table 4. The MVS*_FTO_* showed no significant association with HR_rest_ (β = 0.645, *p* = 0.180). In contrast, significant negative associations were observed for HR_aft_ (β = –4.193, *p* = 0.0004), HR_5min_ (β = –2.055, *p* = 0.0040), and HR_10min_ (β = –1.146, *p* = 0.0087). Significant inverse associations were also observed for ΔHR (β = –4.043, *p* = 0.0012) and HR_max%_ (β = –2.467, *p* = 0.001), along with secondary recovery trajectories at ΔHR_5min_ (β = –1.866, *p* = 0.0170) and ΔHR_10min_ (β = –0.982, *p* = 0.0360). See Table 4 for more details.

### 3.4. Mediation and Interaction Effects of MVS_FTO_, Body Composition, and Physical Activity

In the BMI-mediated model, MVS*_FTO_* showed a significant total effect on HR_aft_ (β = −5.45, *p* < 0.001), with a strong controlled direct effect (β = −13.86, *p* = 0.015) and a modest, nominally significant pure indirect effect via BMI (β = −0.80, *p* = 0.046), indicating partial inconsistent mediation. The larger magnitude of the direct effect relative to the total effect reflects a statistical suppression pattern driven primarily by the positive exposure–mediator interaction component (INT_ref_ = 9.07, *p* = 0.082), which partially attenuates the direct genetic association. For ΔHR, the indirect pathway via BMI was not statistically significant (β = −0.65, *p* = 0.083). For HR_max%_, a modest partial indirect effect was observed through BMI (β = −0.50, *p* = 0.049) alongside a robust total effect (β = −3.29, *p* < 0.001).

In the LTPA-mediated models, total and direct effects of MVS*_FTO_* across all three heart rate outcomes remained robust and statistically significant, while all indirect pathways through LTPA were non-significant (HR_aft_: *p* = 0.108; ΔHR: *p* = 0.111; HR_max%_: *p* = 0.123). Detailed four-way decomposition estimates are provided in Table 5.

In fully adjusted multivariable factorial regression models, potential statistical interaction terms revealed no significant two-way moderation effects for either MVS*_FTO_* × BMI or MVS*_FTO_* × LTPA across the post-exercise heart rate outcomes (all *p* > 0.05). Similarly, exploratory three-way interaction terms (MVS*_FTO_* × BMI × LTPA) did not reach statistical significance in adjusted models, indicating that the observed associations of MVS*_FTO_* with acute exercise heart rate reactivity operate consistently across different adiposity and physical activity strata.

SEM revealed positive direct paths from MVS*_FTO_* to BMI (β = 0.84, *p* = 0.006) and LTPA (β = 188.52, *p* = 0.026). BMI did not predict HR_aft_ (*p* = 0.221), whereas LTPA showed a robust inverse effect (β = −0.0031, *p* < 0.001). The direct effect of MVS*_FTO_* on HR_aft_ remained significant (β = −4.58, *p* = 0.001).

Similar patterns were observed for ΔHR: LTPA was a significant predictor (β = −0.0025, *p* < 0.001), BMI was not (*p* = 0.879), and the direct effect of MVS*_FTO_* persisted (β = −4.04, *p* = 0.001).

**Figure 4:**
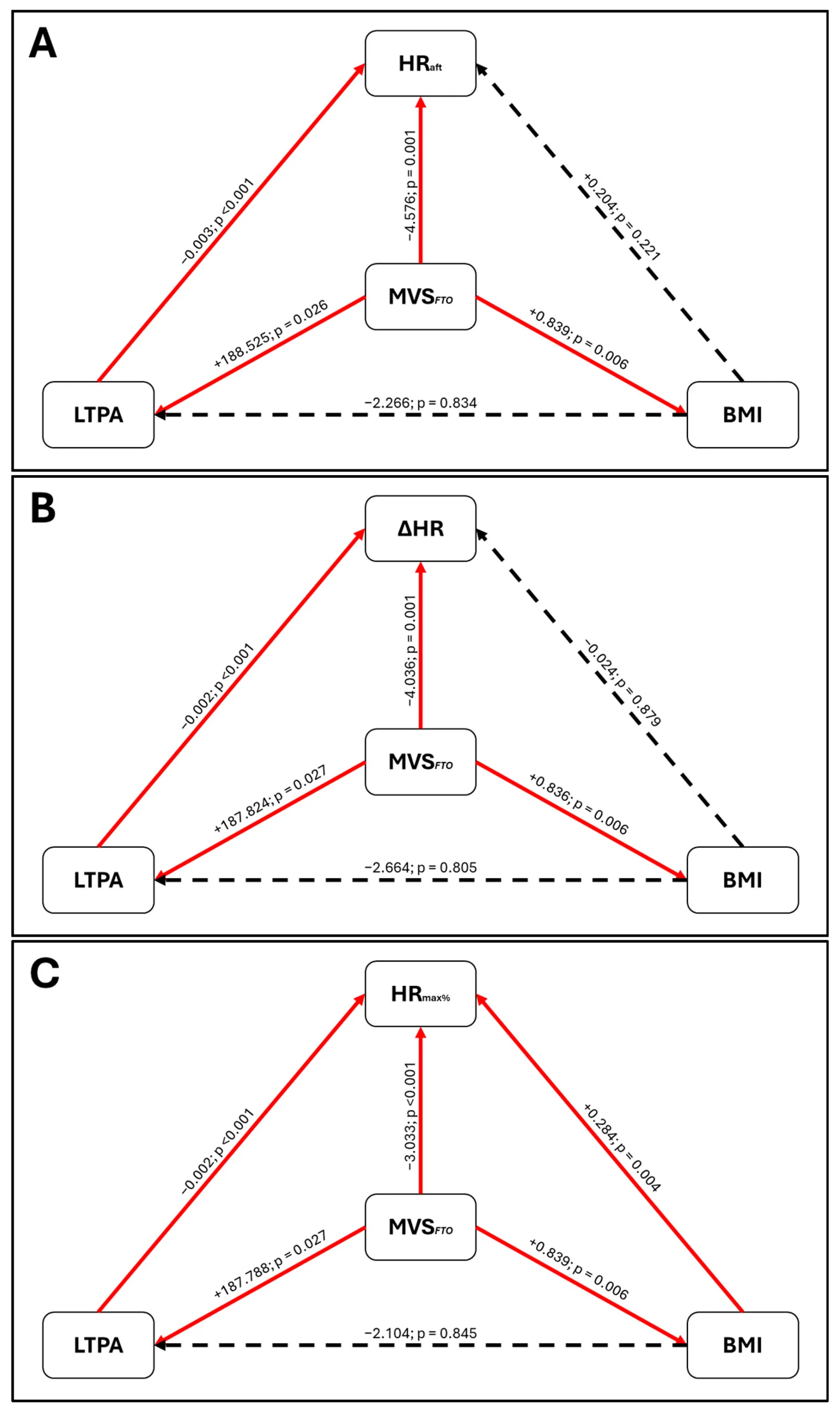
Structural Equation Models of the Effects of Physical Activity, Body Mass Index, and Multi-variant score for *FTO* on Heart Rate Related Outcomes. Panel (**A**) HR_aft_ (first-minute post-exercise heart rate), panel (**B**) ΔHR (delta heart rate), and panel (**C**) HR_max%_ (percentage of predicted maximum heart rate). Red solid arrows indicate statistically significant structural paths (*p* < 0.05), while black dashed arrows represent non-significant pathways. Path coefficients (β) and corresponding *p*-values are shown along each arrow. All models were saturated (df = 0), serving as multivariate path-analytic parameter estimation tools across the observed system.

For HR_max%_, both LTPA (β = −0.002, *p* < 0.001) and BMI (β = 0.284, *p* = 0.004) were significant, while the direct effect of MVS*_FTO_* remained robust (β = −3.03, *p* = 0.001). All SEM models were saturated (df = 0), therefore goodness-of-fit indices (RMSEA, SRMR) are not informative; SEM was utilized strictly for multivariate parameter estimation across the network (Figure 4, with complete parameter estimates detailed in Supplementary Table S7).

### 3.5. Results of Cluster Analyses

Three latent clusters were identified based on three indicators, namely MVS*_FTO_*, BMI, and LTPA. Cluster 1: Low-MVS*_FTO_* profile with high physical activity (*n* = 247); Cluster 2: Intermediate genetic profile with low physical activity (*n* = 140); Cluster 3: High-MVS*_FTO_* profile with moderate physical activity (*n* = 273).

All three cluster-forming indicators differed significantly across clusters (*p* < 0.001). Pairwise comparisons showed significance for all contrasts in MVS*_FTO_* and BMI, except between Clusters 1 and 3 (*p* = 0.052). LTPA was significantly lower in the intermediate-genetic/low-activity cluster (Cluster 2) compared to both the high- and low-MVS_FTO_ clusters (*p* < 0.001), with nominal significance between Clusters 1 and 3 (*p* = 0.014).

No significant differences were observed in Roma ethnicity, sex distribution, or educational attainment across clusters. All pairwise comparisons for these variables were non-significant.

Age differed significantly across clusters (*p* = 0.003), with the intermediate-genetic/low-activity cluster (Cluster 2) being older than both Cluster 1 (*p* = 0.007) and Cluster 3 (*p* = 0.004). No difference was observed between Clusters 1 and 3 (*p* = 1.000).

Waist-to-height ratio showed significant differences across clusters (*p* < 0.001), with nominal significance between Clusters 1 and 3 (*p* = 0.021). Systolic and diastolic blood pressure values were significantly higher in Cluster 2 compared to the other two (*p* < 0.001), while comparisons between Clusters 1 and 3 were not significant.

Domestic physical activity did not differ significantly across clusters (*p* = 0.097), and all pairwise comparisons were non-significant.

GHQ-12 Likert scores were comparable across clusters (*p* = 0.956), with no significant pairwise differences. For more details see Supplementary Table S6.

Kruskal–Wallis tests revealed no significant differences in HR_rest_ across clusters (*p* = 0.086). None of the pairwise comparisons reached nominal or significant thresholds.

HR_aft_ differed significantly across clusters (*p* < 0.001). Pairwise comparisons showed significance between Clusters 2 and 3 (*p* < 0.001), and nominal significance between Clusters 1 and 3 (*p* = 0.041). HR_5min_ showed significant differences across clusters (*p* = 0.001). Pairwise comparison between Clusters 2 and 3 was significant (*p* = 0.001); other comparisons were not significant. HR_10min_ differed significantly across clusters (*p* = 0.002). The comparison between Clusters 2 and 3 was significant (*p* = 0.002); other comparisons were not significant.

ΔHR differed significantly across clusters (*p* = 0.007). Pairwise comparisons between Clusters 2 and 3 (*p* = 0.014) and Clusters 1 and 3 (*p* = 0.049) were both nominally significant. ΔHR_5min_ showed nominal significance across clusters (*p* = 0.036). No pairwise comparisons reached nominal or significant thresholds. ΔHR_10min_ showed nominal significance across clusters (*p* = 0.019). The comparison between Clusters 1 and 3 was nominally significant (*p* = 0.035); other comparisons were not significant.

HR_max%_ differed significantly across clusters (*p* < 0.001). Pairwise comparisons showed significance between Clusters 2 and 3 (*p* < 0.001), and nominal significance between Clusters 1 and 2 (*p* = 0.027) and Clusters 1 and 3 (*p* = 0.041). Overall, these phenotypic cluster profiles were consistent with the multivariable regression models, demonstrating concordant multi-trait patterns across physical activity and multi-variant locus burden tiers. For more details see Table 6.

## 4. Discussion

In this population-based cohort, common *FTO* polymorphisms and an exploratory multi-variant locus score were consistently associated with heart rate responses to submaximal exercise. These associations were robust across HR_aft_, ΔHR, and HR_max%_, after multivariable adjustment for measured adiposity indices and habitual physical activity, while no relationship was observed with resting HR. Importantly, stratified sensitivity analyses demonstrated directionally consistent inverse associations between MVS*_FTO_* and acute exercise heart rate reactivity across both the Hungarian general population (β = −2.65, *p* = 0.051) and Roma minority (β = −3.94, *p* = 0.056) sample groups; while of borderline statistical significance due to subgroup sample sizes, these consistent patterns help reduce concern that the pooled associations were primarily driven by population stratification. While these multivariable models indicate that the observed associations persist independently of measured BMI and self-reported physical activity levels, this statistical persistence should not be conflated with proven biological independence or causal mechanisms given the cross-sectional study design. Downstream exploratory analyses indicated that BMI exhibited only modest partial mediation for HR_aft_ and HR_max%_, whereas LTPA did not mediate any outcome. These path models describe statistical associations rather than proven causal mechanisms or confirmatory biological independence.

Although *FTO* variants are best known for their association with adiposity, body mass index, and metabolic syndrome, emerging evidence suggests broader physiological and cardiovascular relevance. *FTO* is prominently expressed in central nervous system regions—particularly hypothalamic nuclei involved in energy balance, nutrient sensing, and sympathetic/parasympathetic autonomic control [13]. Physical exertion has been shown to rapidly modulate *FTO*-related transcriptional and epitranscriptomic pathways in peripheral tissues such as skeletal muscle and subcutaneous adipose tissue [34,35]. Experimental studies have linked *FTO* activity to fundamental cellular processes, including RNA demethylation, endothelial cell signaling, reactive oxygen species generation, oxidative stress regulation, and skeletal muscle substrate oxidation, providing a plausible biological context for the observed exercise-induced associations [36].

At the molecular level, *FTO* functions primarily as an alpha-ketoglutarate-dependent dioxygenase that catalyzes the reversible demethylation of N^6^-methyladenosine in mRNA and non-coding RNAs [36]. Through this post-transcriptional m^6^A modification, *FTO* dynamically governs mRNA stability, splicing efficiency, and translation rates of key downstream target genes involved in cardiac and vascular homeostasis [37]. Global or cardiac-specific *FTO* deficiency leads to marked alterations in autonomic modulation, characterized by higher resting HR, diminished vagal nerve activity, an elevated low-frequency to high-frequency (LF/HF) ratio, and increased susceptibility to stress-induced tachyarrhythmias and electrophysiological instability [38,39]. Furthermore, m6A demethylation by *FTO* directly regulates the expression of critical cardiac ion channels (such as Na^+^ and Ca^2+^) and intracellular calcium-handling proteins (e.g., SERCA2a and RyR2) [40], which dictate myocardial contractility, action potential duration, and acute chronotropic reactivity. *FTO* also modulates endothelial nitric oxide synthase (eNOS) activation and vascular smooth muscle tone, influencing exercise-induced peripheral vasodilation and cardiac overload [41]. Together with genome-wide association study evidence showing BMI-adjusted associations between *FTO* variants and acute coronary events or vascular dysfunction [42], these molecular insights support the hypothesis that FTO-mediated epitranscriptomic networks may shape acute cardiovascular dynamics under physiological exertion.

Recent epitranscriptomic and translational research further supports the concept that *FTO*-related pathways influence cardiovascular reactivity across multiple interconnected biological axes. *FTO* acts as a context-dependent master regulator integrating metabolic remodeling, immuno-inflammatory cascade signaling [43], and cardiac structural pathways [37]. These cellular axes encompass glycolytic flux regulation, mitochondrial oxidative phosphorylation and ATP generation [44], pro-inflammatory cytokine expression (e.g., TNF-alpha, IL-6), and extracellular matrix remodeling in cardiac tissue [43]. During physical exercise, rapid metabolic stress triggers transient alterations in intracellular metabolite levels (alpha-ketoglutarate, succinate) [45], which may directly alter *FTO* enzymatic activity. This metabolic–enzymatic feedback loop influences how cardiac and vascular tissues respond to acute catecholaminergic stimulation during submaximal effort. Although these precise intracellular mechanisms were not directly measured in our observational cohort, they establish a coherent molecular framework wherein *FTO* genetic variation influences acute exercise HR dynamics beyond adiposity-related pathways.

Our findings align with prior reports of context-dependent *FTO* effects. In the Women’s Genome Health Study, the rs8050136 variant increased cardiovascular risk primarily among physically inactive women [46], highlighting the modifying role of lifestyle factors. It is noteworthy that rs9939609 (the most frequently studied *FTO* variant) showed the weakest associations with exercise-related HR traits in our cohort. This may help explain why a previous study reported null findings [47], as examining a single variant captures only part of the genetic signal within the high-LD region. In contrast, variants such as rs1121980 and rs1558902, and especially the cumulative allelic load score, showed substantially stronger and more consistent effects. In the OPERA cohort, the rs9939609 AA genotype predicted long-term cardiovascular risk even after adjustment for BMI [48]. Importantly, the context-dependent nature of *FTO* effects also implies that variants showing robust associations with chronic cardiometabolic outcomes (such as rs9939609) may not necessarily be the strongest predictors of acute exercise-related HR dynamics.

Notably, the *FTO* locus was not identified in the UK Biobank GWAS of exercise HR response and recovery [49]. Differences in exercise modality (maximal vs. submaximal), phenotype definition, population structure, and environmental exposures may explain this discrepancy. Therefore, our findings should be considered hypothesis-generating and warrant replication in independent cohorts using harmonized protocols.

Exploratory structural equation modeling indicated modest partial mediation by BMI and no consistent mediation by LTPA. The predominant direct statistical paths suggest that the MVS*_FTO_* captures associations with HR responses that remain robust after accounting for measured body mass or habitual activity, supporting an inconsistent mediation framework rather than establishing causal autonomy.

Regarding effect magnitude, each additional coded allele was associated with a modest decrease of approximately 3.6 to 4.2 beats per minute in first-minute post-exercise heart rate and net acute reactivity. Within the context of our cross-sectional study design, this ~4 bpm difference should not be overstated as evidence of major clinical impact, public health relevance, or superior cardiovascular fitness. Instead, it represents an exploratory physiological observation, a subtle piece of a complex cardiometabolic mosaic, indicating that genetic variation at the *FTO* locus may contribute to modest inter-individual variations in acute chronotropic reactivity to submaximal exercise. Furthermore, because our cross-sectional study did not evaluate longitudinal training responses, exercise adaptations, or the predictive accuracy of these genetic variants, these observations cannot be directly extrapolated to personalized exercise prescription or tailored activity programming. These findings serve primarily as hypothesis-generating evidence that warrants cautious interpretation and prospective replication in independent cohorts and randomized exercise trials.

While the inverse associations of coded *FTO* alleles with acute post-exercise heart rate reactivity (HR_aft_, ΔHR, and HR_max%_) robustly survived strict Bonferroni correction, observed relationships with extended recovery parameters were evaluated under the FDR procedure as exploratory findings. Because extended recovery kinetics did not uniformly maintain robust associations across all time points under multiple-testing adjustment, they should not be interpreted as confirmatory evidence of altered parasympathetic reactivation kinetics. Instead, they represent preliminary, exploratory trends that require rigorous validation in independent, larger cohorts using continuous heart rate recovery monitoring protocols.

A major strength of the present study is the well-characterized, population-based cohort (*n* = 660) evaluated under a standardized exercise protocol (YMCA 3-min step test), allowing the assessment of acute post-exercise hemodynamic reactivity and recovery kinetics. Methodologically, our investigation employed multiple complementary analytical approaches—including multivariable linear regression, multi-variant locus scoring (MVS*_FTO_*), counterfactual four-way mediation decomposition, and structural equation modelling—alongside extensive sensitivity analyses evaluating potential population stratification across general Hungarian and Roma minority populations. Furthermore, rigorous diagnostic procedures and hierarchical multiple-testing corrections were implemented to minimize Type I error inflation.

Several limitations should be acknowledged. First, a major genetic limitation is the very high pairwise linkage disequilibrium among the four genotyped *FTO* variants (r = 0.948–0.966, D’ = 0.953–0.997). Due to this extensive haplotype block structure, individual single-nucleotide polymorphisms carry largely redundant statistical information. Consequently, the observed cardiovascular associations cannot be attributed to any single causal variant among those tested; rather, they reflect the collective biological signal of the core *FTO* intron-1 locus as a whole. Furthermore, because variant inclusion for the MVS*_FTO_* was partly evaluated using internal model performance on ΔHR, the score must be interpreted strictly as an exploratory summary index of regional locus burden rather than a confirmatory or independently optimized polygenic predictor. Consequently, its association with ΔHR cannot be considered a genuinely confirmatory finding within this same cohort, and prospective validation in independent populations is indispensable before this multi-locus construct can be generalized. Similarly, because both the structural equation modeling and multi-trait latent cluster analyses were performed within the same observational dataset, they represent internal exploratory corroboration rather than independent confirmation of the findings. Second, the cross-sectional study design precludes causal inference or the verification of temporal mediation sequences. Additionally, while our final analytical sample represented 82.8% of the examined cohort (660 of 797 individuals who proceeded to examination), potential selection bias cannot be ruled out entirely. Third, BMI and waist-to-height ratio were utilized as indirect anthropometric proxies for body composition rather than direct compartmental assessments (such as dual-energy X-ray absorptiometry or bioimpedance), and habitual physical activity was assessed using self-reported questionnaires (IPAQ-LF), which are subject to recall and social desirability biases that may leave residual confounding in multivariable models. Fourth, detailed data on heart-rate-modifying medications (e.g., β-blockers or calcium channel blockers) and specific subclinical chronotropic conditions were unavailable, representing a potential source of unmeasured residual confounding. Although we adjusted for all available sociodemographic, lifestyle, anthropometric, and hemodynamic covariates, the influence of unrecorded pharmacological treatments cannot be completely ruled out. Fifth, exercise heart rate dynamics were evaluated via manual radial pulse palpation rather than continuous electrocardiographic or telemetry monitoring, which may underestimate true peak heart rate or subtle early recovery kinetics; however, because such measurement error is non-differential with respect to genotype, it would most likely bias observed associations toward the null rather than generate spurious positive findings. Finally, the regional sampling frame in northeastern Hungary may limit direct generalizability to populations with different ancestral or environmental backgrounds.

## 5. Conclusions

Common *FTO* variants and an exploratory *FTO* multi-variant score were consistently associated with acute heart rate responses to submaximal exercise in this population-based cross-sectional cohort. Higher MVS*_FTO_* values were associated with lower first-minute post-exercise HR and smaller acute HR elevations, indicating an observational genetic link with inter-individual variability in short-term cardiovascular responses to physical activity. These associations remained statistically significant after adjusting for measured BMI, body composition, and habitual physical activity levels. Rather than confirming biological causality, clinical utility, or providing confirmatory evidence from an internally derived score, these observational findings establish a statistical basis for novel metabolic-genetic hypotheses regarding exercise-induced autonomic reactivity. Independent external validation of the exploratory multi-variant score, along with replication in prospective cohorts and controlled exercise trials, is required to verify these findings and elucidate their underlying physiological mechanisms.

## Figures and Tables

**Table 1:** Sociodemographic, physical and behavioral characteristics across YMCA post-exercise heart rate fitness categories.

		Very Poor and Poor (*n =* 191)	Above Average/Average/Below Average (*n =* 295)	Good and Excellent (*n =* 174)	*p* for Trend
		Average in % (95%CI)			
Roma		59.77% (52.37–66.85)	39.66% (34.20–45.32)	58.64% (51.57–65.45)	0.999
Women		67.24% (60.03–73.89)	56.27% (50.57–61.85)	79.06% (72.88–84.37)	0.010 *
Education	Less than primary or primary	60.92% (53.54–67.94)	45.76% (40.14–51.47)	59.16% (52.10–65.95)	0.708
	Vocational or high school	27.59% (21.35–34.56)	43.39% (37.82–49.09)	35.08% (28.58–42.03)	
	College or university	11.49% (7.40–16.86)	10.85% (7.68–14.78)	5.76% (3.10–9.75)	
		Median (interquartile range)			*p* for trend
Age (years)		41.50 (38.00–44.00)	44.00 (42.00–46.00)	46.00 (45.00–50.00)	0.009 *
Height (cm)		160.00 (159.00–164.00)	165.00 (165.00–168.00)	164.00 (164.00–166.00)	0.450
Weight (kg)		71.00 (67.00–78.00)	76.00 (74.00–80.00)	71.00 (70.00–74.00)	0.144
Body Mass Index (kg/m^2^)		27.20 (26.06–28.07)	26.89 (26.29–27.61)	26.03 (24.82–27.01)	0.102
Waist circumference (cm)		97.00 (93.00–100.00)	96.00 (95.00–98.00)	90.00 (89.00–94.00)	<0.001 *
Systolic BP (mmHg)		121.25 (120.00–125.00)	126.00 (125.00–130.00)	125.00 (120.00–128.00)	0.759
Diastolic BP (mmHg)		80.00 (80.00–85.00)	80.00 (80.00–92.50)	80.00 (80.00–82.50)	0.012 *
Domestic Physical Activity (MET-min/week)		1650.00 (1440.00–1890.00)	2160.00 (1980.00–2520.00)	2510.00 (2070.00–2940.00)	<0.001 *
Leisure-Time Physical Activity (MET-min/week)		231.00 (198.00–330.00)	495.00 (360.00–720.00)	495.00 (396.00–819.00)	<0.001 *
GHQ-12 Likert Score		9.50 (9.00–10.00)	10.00 (10.00–11.00)	10.00 (10.00–12.00)	0.272

BP: blood pressure; MET-min/week: metabolic equivalent of task per week; *: *p* < 0.05.

**Table 2:** Incremental model statistics for *FTO* variants considered for inclusion in the multi-variant score (MVS*_FTO_*).

MVS*_FTO_*	β (95% CI)	*p*-Value	Model R^2^	Adj. R^2^	∆ R^2^	Root Mean Squared Error	Contribution to Final MVS*_FTO_* Model
rs1121980 (A)	−3.797 (−6.408–−1.187)	0.0044	0.098	0.081	Reference	23.902	Reference
rs1121980 (A) rs1558902 (A)	−1.959 (−3.277–−0.640)	0.0037	0.099	0.082	0.0005	23.895	rs1558902 included
rs1121980 (A) rs1558902 (A) rs9939609 (A)	−1.305 (−2.190–−0.421)	0.0039	0.099	0.082	−0.0001	23.898	rs9939609 excluded
rs1121980 (A) rs1558902 (A) rs9941349 (C)	−4.043 (−6.489–−1.596)	0.0012	0.102	0.085	0.0028	23.858	rs9941349 included

**Table 3:** Heart Rate Parameters Across *FTO* multi-variant score Groups.

	Multi-Variant Score			*p* for Trend
Heart Rate Parameter	Low (MVS: 1–2) *n =* 218	Intermediate (MVS *=* 3) *n =* 305	High (MVS: 4–6) *n =* 137	
	Average (95%CI)			
Resting heart rate	77.68 (76.27–79.09)	77.59 (76.49–78.69)	77.39 (75.48–79.30)	0.929
Post-exercise heart rate	115.16 (111.40–118.93)	111.56 (108.62–114.51)	104.10 (100.30–107.90)	<0.001 *
Heart rate after 5 min rest	95.93 (93.51–98.35)	93.01 (91.21–94.81)	89.99 (87.37–92.61)	0.003 *
Heart rate after 10 min rest	83.49 (81.91–85.06)	81.83 (80.49–83.18)	80.74 (78.67–82.80)	0.022 *
Delta heart rate	37.48 (33.75–41.21)	33.97 (31.19–36.76)	26.71 (23.49–29.93)	0.001 *
Delta heart rate after 5 min rest	18.25 (15.91–20.60)	15.42 (13.72–17.11)	12.59 (10.56–14.63)	0.006 *
Delta heart rate after 10 min rest	5.81 (4.50–7.12)	4.24 (3.16–5.33)	3.34 (2.24–4.45)	0.029 *
Percent of predicted maximum heart rate	65.37 (63.26–67.48)	63.68 (61.99–65.38)	58.71 (56.07–61.35)	<0.001 *

Values are presented as unadjusted mean with 95% confidence intervals across unweighted multi-variant score (MVS*_FTO_*) tiers. *p*-values for linear trend across genetic burden categories were evaluated using the non-parametric Jonckheere–Terpstra trend test. *: significant after accounting for multiple comparisons within the analytical family (strict threshold *p* < 0.0125 for primary reactivity traits [post-exercise heart rate, delta heart rate, and percent of predicted maximum heart rate]; FDR-adjusted q < 0.05 for secondary recovery traits [heart rate after 5- or 10-min rest, and delta heart rate after 5- or 10-min rest]).

**Table 4:** Associations between the *FTO* multi-variant score and Heart Rate Parameters.

	β (95% CI)	*p*-Value	Model R^2^	Adj. R^2^	Root MSE
Resting heart rate	0.645 (−0.291–1.582)	0.180	0.228	0.213	9.044
Post-exercise heart rate	−4.193 (−6.515–−1.870)	0.0004 *	0.264	0.249	22.622
Heart rate after 5 min rest	−2.055 (−3.454–−0.656)	0.0040 *	0.334	0.321	13.616
Heart rate after 10 min rest	−1.146 (−2.000–−0.291)	0.0087 *	0.519	0.509	8.323
Delta heart rate	−4.043 (−6.489–−1.596)	0.0012 *	0.102	0.085	23.858
Delta heart rate after 5 min rest	−1.866 (−3.394–−0.339)	0.0170 *	0.077	0.060	14.883
Delta heart rate after 10 min rest	−0.982 (−1.901–−0.063)	0.0360 *	0.046	0.028	8.962
Percent of predicted maximum heart rate	−2.467 (−3.908–−1.025)	0.0010 *	0.204	0.189	14.058

β: β estimates, 95%CI: 95% Confidence Intervals, and *p*-values. *: significant after accounting for multiple comparisons within the analytical family (strict threshold *p* < 0.0125 for primary reactivity traits [post-exercise heart rate, delta heart rate, and percent of predicted maximum heart rate]; FDR-adjusted q < 0.05 for secondary recovery traits [heart rate after 5- or 10-min rest, and delta heart rate after 5- or 10-min rest]).

**Table 5:** Mediation Models: Four-Way Decomposition of multi-variant score for *FTO* on Post-Exercise HR Outcomes.

Outcome	Mediator	Total Effect (TE)	Controlled Direct Effect (CDE)	Reference Interaction (INT_ref_)	Mediated Interaction (INT_med_)	Pure Indirect Effect (PIE)	Mediation Type
				β (95%CI), *p*-Value			
HR_aft_	BMI	−5.45 (−8.05–−2.85) *p* < 0.001 *	−13.86 (−24.99–−2.73) *p =* 0.015 *	9.07 (−1.15–19.29) *p* = 0.082	0.15 (−0.05–0.35) *p* = 0.142	−0.80 (−1.59–−0.01) *p =* 0.046 *	Partial
	LTPA	−5.52 (−8.38–−2.67) *p* < 0.001 *	−5.91 (−8.73–−3.09) *p* < 0.001 *	1.15 (−0.14–2.44) *p* = 0.080	0.21 (−0.09–0.52) *p* = 0.166	−0.98 (–2.19–0.23) *p =* 0.108	None
ΔHR	BMI	−5.09 (−7.79–−2.39) *p* < 0.001 *	−12.47 (−24.22–−0.73) *p =* 0.037 *	8.16 (−1.82–18.14) *p* = 0.109	0.13 (−0.06–0.33) *p* = 0.181	−0.65 (−1.39–0.08) *p =* 0.083	None
	LTPA	−5.42 (−8.41–−2.42) *p* < 0.001 *	−5.84 (−8.81–−2.86) *p* < 0.001 *	1.20 (−0.15–2.55) *p* = 0.082	0.22 (−0.09–0.54) *p* = 0.161	−1.00 (−2.22–0.23) *p =* 0.111	None
HR_max%_	BMI	−3.29 (−4.93–−1.64) *p* < 0.001 *	−6.56 (−13.60–0.48) *p =* 0.068	3.82 (−1.94–9.58) *p* = 0.193	0.05 (−0.07–0.17) *p* = 0.380	−0.50 (−1.01–−0.00) *p =* 0.049 *	Partial
	LTPA	−3.27 (−5.01–−1.54) *p* < 0.001 *	−3.33 (−5.12–−1.54) *p* < 0.001 *	0.53 (−0.30–1.36) *p* = 0.210	0.06 (−0.11–0.24) *p* = 0.473	−0.53 (−1.20–0.14) *p =* 0.123	None

This table summarizes the results of mediation models using the counterfactual four-way decomposition approach (med4way), examining the effect of the cumulative *FTO* multi-variant score (MVS*_FTO_*) on post-exercise heart rate parameters (HR_aft_, ΔHR, and HR_max%_). Mediators are Body Mass Index (BMI) and Leisure-Time Physical Activity (LTPA). Models report Total Effect (TE), Controlled Direct Effect (CDE), Reference Interaction (INT_ref_), Mediated Interaction (INT_med_) and Pure Indirect Effect (PIE) alongside 95% confidence intervals and *p*-values. In the four-way decomposition framework (TE = CDE + INT_ref_ + INT_med_ + PIE), the larger magnitude of CDE relative to TE reflects an inconsistent mediation/suppression pattern driven by the exposure-mediator interaction component. *: statistically significant under the exploratory evaluation framework (*p* < 0.05).

**Table 6:** Heart Rate Parameters Across Cluster Groups.

	Cluster 1: Low-MVS*_FTO_* Profile with High Physical Activity (*n* = 247)	Cluster 2: Intermediate Genetic Profile with Low Physical Activity (*n* = 140)	Cluster 3: High-MVS*_FTO_* Profile with Moderate Physical Activity (*n* = 273)	*p* for Trend ^a^	Corrected *p*-Value ^b^		
	Average (95%CI)				1 vs. 2	2 vs. 3	1 vs. 3
Resting heart rate	77.18 (75.88–78.49)	78.64 (77.06–80.22)	77.39 (76.10–78.67)	0.086	0.129	0.137	1.000
Post-exercise heart rate	112.96 (109.54–116.38)	115.56 (111.19–119.93)	107.34 (104.32–110.36)	<0.001 *	0.191	<0.001 *	0.041 *
Heart rate after 5 min rest	94.31 (92.13–96.48)	96.09 (93.31–98.87)	91.09 (89.18–93.00)	0.001 *	0.400	0.001 *	0.060
Heart rate after 10 min rest	82.52 (81.08–83.96)	84.06 (82.21–85.92)	80.84 (79.32–82.36)	0.002 *	0.348	0.002 *	0.084
Delta heart rate	35.78 (32.43–39.14)	36.92 (32.49–41.36)	29.95 (27.30–32.61)	0.007 *	1.000	0.014 *	0.049 *
Delta heart rate after 5 min rest	17.13 (15.04–19.21)	17.45 (14.64–20.26)	13.70 (12.09–15.31)	0.036 *	1.000	0.077	0.111
Delta heart rate after 10 min rest	5.34 (4.16–6.52)	5.42 (3.79–7.06)	3.45 (2.43–4.48)	0.019 *	1.000	0.097	0.035 *
Percent of predicted maximum heart rate	63.70 (61.77–65.64)	67.78 (65.23–70.34)	60.37 (58.55–62.20)	<0.001 *	0.027 *	<0.001 *	0.041 *

Values are presented as mean (95% confidence interval). ^a^: *p*-values for ordered trend across clusters were evaluated using the non-parametric Jonckheere–Terpstra trend test. ^b^: pairwise comparisons with Bonferroni adjustment following Kruskal–Wallis tests. *: statistically significant after accounting for multiple comparisons (strict threshold *p* < 0.0125 for primary reactivity traits [post-exercise heart rate, delta heart rate, and percent of predicted maximum heart rate]; FDR-adjusted q < 0.05 for secondary recovery traits [heart rate after 5- or 10-min rest, and delta heart rate after 5- or 10-min rest]; adjusted *p* < 0.05 for pairwise post hoc comparisons).

## Data Availability

Due to data protection and ethical concerns, the datasets supporting the conclusions of this article are available upon request from the study coordinators, Roza Adány (adany.roza@med.unideb.hu) and Peter Piko (piko.peter@med.unideb.hu).

## Supplementary Materials

The following supporting information can be downloaded at https://www.mdpi.com/article/10.3390/biology15181607/s1, Figure S1: Flowchart of the creation of study sample. Figure S2: Association of FTO SNPs with resting heart rate. The figure shows the β-values (in beats per minute) and the 95% confidence intervals obtained from the linear regression analyses. Figure S3: Association of FTO SNPs with post-exercise heart rate after 5 min rest. The figure shows the β-values (in beats per minute) and the 95% confidence intervals obtained from the linear regression analyses. *: False discovery rate-adjusted significance (q < 0.05). Figure S4: Association of FTO SNPs with post-exercise heart rate after 10 min rest. The figure shows the β-values (in beats per minute) and the 95% confidence intervals obtained from the linear regression analyses. *: False discovery rate-adjusted significance (q < 0.05). Figure S5: Association of FTO SNPs with delta heart rate after 5 min rest. The figure shows the β-values (in beats per minute) and the 95% confidence intervals obtained from the linear regression analyses. Figure S6: Association of FTO SNPs with delta heart rate after 10 min rest. The figure shows the β-values (in beats per minute) and the 95% confidence intervals obtained from the linear regression analyses. Table S1: Allele coding, genotype distributions, Hardy–Weinberg equilibrium, and association directions across single-SNP and full multi-SNP regression models for the analyzed FTO variants. Table S2: Pairwise linkage disequilibrium between the four FTO SNPs (n = 654). Table S3: Age- and sex-specific post-exercise heart rate cut-offs for YMCA step test classification. Table S4: Sensitivity analysis of individual FTO variants with immediate post-exercise heart rate using sequential adjustment models. Table S5: Sociodemographic, physical and behavioral characteristics across FTO-related multi-variant score (MVSFTO) groups. Table S6: Sociodemographic, physiological, and behavioral characteristics across latent clusters identified by hierarchical analysis. Table S7: Complete structural equation model (SEM) path coefficients and parameter estimates for post-exercise heart rate traits.

## Abbreviations

The following abbreviations are used in this manuscript:

BMI	body mass index
BP	blood pressure
CV	cardiovascular
EHES	European Health Examination Survey
EHIS	European Health Interview Survey
*FTO*	fat mass and obesity-associated
GHQ-12	12-item General Health Questionnaire
HR	heart rate
HRR	heart rate recovery
IPAQ-LF	International Physical Activity Questionnaire—Long Form
LD	linkage disequilibrium
LTPA	leisure-time physical activity
MET	metabolic equivalent of task
MVS*_FTO_*	*FTO*-related multi-variant score
SEM	structural equation modelling
SNP	single nucleotide polymorphism
WHtR	waist-to-height ratio
YMCA	Young Men’s Christian Association
