# Supplementary material for: Associations Between FTO (Fat Mass- and Obesity-Associated) Gene Allelic Load and Heart Rate Responses to Submaximal Exercise in Humans: A Cross-Sectional Study
Source: Biology (Basel). 2026 Sep 11;15(18):1607. doi: 10.3390/biology15181607 (PMC13604555; doi:10.3390/biology15181607)
Supplement: Supplementary file 1 [file biology-15-01607-s001.zip › Supplementary Figure S4.pdf]

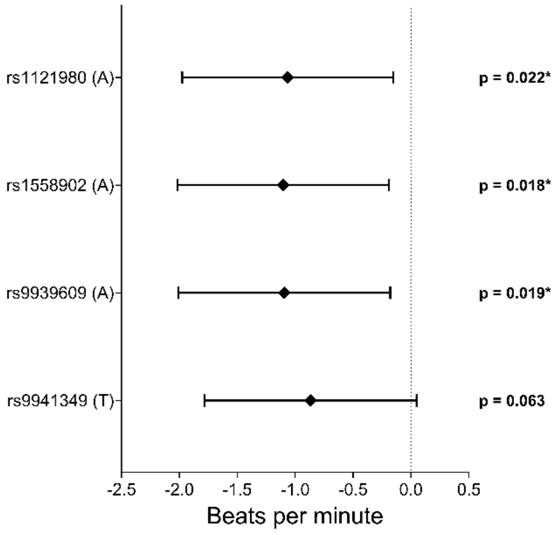

Supplementary Figure S4. Association of *FTO* SNPs with post-exercise heart rate after 10 minutes rest. The figure shows the β-values (in beats per minute) and the 95% confidence intervals obtained from the linear regression analyses. *: False discovery rate-adjusted significance ($q < 0.05$)
