# Supplementary material for: Associations Between FTO (Fat Mass- and Obesity-Associated) Gene Allelic Load and Heart Rate Responses to Submaximal Exercise in Humans: A Cross-Sectional Study
Source: Biology (Basel). 2026 Sep 11;15(18):1607. doi: 10.3390/biology15181607 (PMC13604555; doi:10.3390/biology15181607)
Supplement: Supplementary file 1 [file biology-15-01607-s001.zip › Supplementary Table S1.pdf]

Supplementary Table S1. Allele coding, genotype distributions, Hardy–Weinberg equilibrium, and association directions across single-SNP and full multi-SNP regression models for the analyzed *FTO* variants.

SNP ID	Ref/Coded Allele	Obesity Risk Allele	Genotype Coding	Genotype Counts (n, %)	Coded Allele Frequency (MAF)	HWE p-value	Single-SNP Direction	Full Multi-SNP Direction
rs1558902	T / A	A	T/T = 0<br>T/A = 1<br>A/A = 2	T/T: 221 (33%)<br>T/A: 314 (48%)<br>A/A: 125 (19%)	43%	p = 0.470	Negative	Negative
rs1121980	G / A	A	G/G = 0<br>G/A = 1<br>A/A = 2	G/G: 210 (32%)<br>G/A: 316 (48%)<br>A/A: 134 (20%)	44%	p = 0.480	Negative	Negative
rs9939609	T / A	A	T/T = 0<br>T/A = 1<br>A/A = 2	T/T: 224 (34%)<br>T/A: 314 (48%)<br>A/A: 122 (18%)	42%	p = 0.520	Negative	Negative
rs9941349	C / T	T	C/C = 0<br>C/T = 1<br>T/T = 2	C/C: 223 (34%)<br>C/T: 313 (47%)<br>T/T: 124 (19%)	42%	p = 0.470	Negative	Positive

Ref = Reference allele; Coded = Effect allele parameterized in the linear additive model; MAF = Minor allele frequency. Single-SNP Direction: Derived from models testing each SNP individually. Negative direction indicates lower delta heart rate. Full Multi-SNP Direction: Derived from a model entering all four SNPs simultaneously with all covariates. The positive shift for rs9941349 in the full model reflects a parameter suppression effect caused by high linkage disequilibrium ($r > 0.94$, VIF = 15.72), justifying composite score construction over simultaneous entry.
