# Supplementary material for: Associations Between FTO (Fat Mass- and Obesity-Associated) Gene Allelic Load and Heart Rate Responses to Submaximal Exercise in Humans: A Cross-Sectional Study
Source: Biology (Basel). 2026 Sep 11;15(18):1607. doi: 10.3390/biology15181607 (PMC13604555; doi:10.3390/biology15181607)
Supplement: Supplementary file 1 [file biology-15-01607-s001.zip › Supplementary Table S2.pdf]

**Supplementary Table S2.** Pairwise linkage disequilibrium between the four *FTO* SNPs (n=654)

| SNP1 | SNP2 | D′ | r |
|---|---|---|---|
| rs1121980 | rs1558902 | 0.9967 | 0.9665 |
| rs1121980 | rs9939609 | 0.9902 | 0.9512 |
| rs1121980 | rs9941349 | 0.9902 | 0.9557 |
| rs1558902 | rs9939609 | 0.9684 | 0.9594 |
| rs1558902 | rs9941349 | 0.9528 | 0.9483 |
| rs9939609 | rs9941349 | 0.9685 | 0.9640 |
