# Supplementary material for: Associations Between FTO (Fat Mass- and Obesity-Associated) Gene Allelic Load and Heart Rate Responses to Submaximal Exercise in Humans: A Cross-Sectional Study
Source: Biology (Basel). 2026 Sep 11;15(18):1607. doi: 10.3390/biology15181607 (PMC13604555; doi:10.3390/biology15181607)
Supplement: Supplementary file 1 [file biology-15-01607-s001.zip › Supplementary Table S3.pdf]

Supplementary Table S3. Age- and sex-specific post-exercise heart rate cut-offs for YMCA step test classification.

Women	Age groups (in years)				
	20 – 25	26 – 35	36 – 45	46 – 55	56 – 64
	Range of bpm				
Good/Excellent	52 – 95	58 – 94	51 – 99	63 – 109	60 – 105
Average	96 – 120	95 – 121	100 – 123	110 – 125	106 – 128
Very poor/Poor	121 – 169	122 – 171	124 – 169	126 – 171	129 – 174
**Men**	Age groups (in years)				
	20 – 25	26 – 35	36 – 45	46 – 55	56 – 64
	Range of bpm				
Good/Excellent	50 – 87	51 – 87	49 – 91	56 – 94	60 – 96
Average	88 – 110	88 – 113	92 – 115	95 – 120	97 – 118
Very poor/Poor	111 – 157	114 – 161	116 – 163	121 – 159	119 – 154

Values represent 1-minute post-exercise recovery heart rate ranges in beats per minute (bpm) according to the standardized YMCA 3-minute step test normative protocols. Original categories (Excellent, Good, Above Average, Average, Below Average, Poor, Very Poor) were collapsed into three mutually exclusive, age- and sex-specific fitness tiers.
