# Supplementary material for: Associations Between FTO (Fat Mass- and Obesity-Associated) Gene Allelic Load and Heart Rate Responses to Submaximal Exercise in Humans: A Cross-Sectional Study
Source: Biology (Basel). 2026 Sep 11;15(18):1607. doi: 10.3390/biology15181607 (PMC13604555; doi:10.3390/biology15181607)
Supplement: Supplementary file 1 [file biology-15-01607-s001.zip › Supplementary Table S4.pdf]

Supplementary Table S4. Sensitivity analysis of individual *FTO* variants with immediate post-exercise heart rate using sequential adjustment models

Modell	rs1121980	rs1558902	rs9939609	rs9941349
	Coefficient (95%CI) - p-value			
Model 1 - crude	-4.94 (-7.73 – -2.15) p=0.001	-5.00 (-7.80 – -2.19) p=0.001	-5.03 (-7.84 – -2.22) p=0.001	-4.32 (-7.13 – -1.51) p=0.003
Model 2	-4.78 (-7.56 – -2.01) p=0.001	-4.86 (-7.64 – -2.07) p=0.001	-4.92 (-7.71 – -2.12) p=0.001	-4.26 (-7.05 – -1.47) p=0.003
Model 3	-4.65 (-7.42 – -1.88) p=0.001	-4.69 (-7.46 – -1.91) p=0.001	-4.75 (-7.53 – -1.98) p=0.001	-4.09 (-6.87 – -1.31) p=0.004
Model 4	-4.73 (-7.44 – -2.02) p=0.001	-4.71 (-7.43 – -1.99) p=0.001	-4.70 (-7.43 – -1.98) p=0.001	-4.19 (-6.92 – -1.46) p=0.003
Model 5 - full	-4.11 (-6.60 – -1.63) p=0.001	-4.18 (-6.67 – -1.70) p=0.001	-4.11 (-6.61 – -1.62) p=0.001	-3.55 (-6.05 – -1.06) p=0.005

Notes: β-coefficients (95% CI) and p-values are shown. Models are adjusted sequentially as follows: Model 1 (Crude), Model 2 (Demographics: ethnicity, sex, age), Model 3 (Model 2 + Adiposity: BMI, WHtR), Model 4 (Model 3 + CVR: smoking, systolic and diastolic blood pressure), Model 5 (Model 4 + Lifestyle/Psychosocial: LTPA, Domestic physical activity, education, GHQ-12).
