# Supplementary material for: Associations Between FTO (Fat Mass- and Obesity-Associated) Gene Allelic Load and Heart Rate Responses to Submaximal Exercise in Humans: A Cross-Sectional Study
Source: Biology (Basel). 2026 Sep 11;15(18):1607. doi: 10.3390/biology15181607 (PMC13604555; doi:10.3390/biology15181607)
Supplement: Supplementary file 1 [file biology-15-01607-s001.zip › Supplementary Table S5.pdf]

Supplementary Table S5. Sociodemographic, physical and behavioral characteristics across *FTO*-related multi-variant score (MVS_{FTO}) groups.

		Multi-variant score categories			
		Low (MVS: 1 – 2) n=218	Intermediate (MVS=3) n=305	High (MVS: 4 – 6) n=137	p for trend
		Prevalence in % (95%CI)			
Roma		51.83 (45.21 – 58.41)	47.87 (42.30 – 53.47)	54.01 (45.66 – 62.20)	0.898
Women		67.43 (61.01 – 73.39)	65.90 (60.45 – 71.05)	62.77 (54.48 – 70.53)	0.388
Education	Less than primary or primary	56.88 (50.25 – 63.33)	51.48 (45.87 – 57.05)	53.28 (44.93 – 61.50)	0.271
	Vocational or high school	36.24 (30.07 – 42.77)	37.38 (32.09 – 42.91)	36.50 (28.78 – 44.77)	
	College or university	6.88 (4.08 – 10.82)	11.15 (7.98 – 15.04)	10.22 (5.98 – 16.12)	
		Average (95%CI)			p for trend
Age (years)		43.55 (41.81 – 45.28)	43.44 (42.11 – 44.78)	42.03 (39.89 – 44.17)	0.294
Body Mass Index (kg/m²)		26.66 (25.94 – 27.37)	26.96 (26.27 – 27.65)	28.75 (27.64 – 29.86)	0.015*
Waist-to-Height Ratio		0.57 (0.56–0.59)	0.57 (0.56 – 0.58)	0.59 (0.57 – 0.61)	0.384
Systolic BP (mmHg)		125.30 (123.18 – 127.41)	125.18 (123.32 – 127.03)	125.41 (122.73 – 128.10)	0.782
Diastolic BP (mmHg)		78.92 (77.69 – 80.16)	79.57 (78.48 – 80.65)	79.03 (77.53 – 80.53)	0.993
Domestic Physical Activity (MET-min/week)		2,805.11 (2,462.65 – 3,147.58)	3,076.55 (2,738.59 – 3,414.52)	2,994.91 (2,468.15 – 3,521.67)	0.916
Leisure-Time Physical Activity (MET-min/week)		976.54 (771.10 – 1,181.97)	1,187.84 (979.75 – 1,395.92)	1,511.12 (1,167.83 – 1,854.42)	0.028*
GHQ-12 Likert Score		10.82 (10.21 – 11.43)	10.48 (9.95 – 11.02)	11.25 (10.32 – 12.18)	0.883

BP: blood pressure; MET-min/week: Metabolic Equivalent of Task per Week; *: p <0.05
