# Supplementary material for: Associations Between FTO (Fat Mass- and Obesity-Associated) Gene Allelic Load and Heart Rate Responses to Submaximal Exercise in Humans: A Cross-Sectional Study
Source: Biology (Basel). 2026 Sep 11;15(18):1607. doi: 10.3390/biology15181607 (PMC13604555; doi:10.3390/biology15181607)
Supplement: Supplementary file 1 [file biology-15-01607-s001.zip › Supplementary Table S6.pdf]

**Supplementary Table S6.** Sociodemographic, physiological, and behavioral characteristics across latent clusters identified by hierarchical analysis

| | | Cluster 1: Low-$MVS_{FTO}$ profile with high physical activity (n = 247) | Cluster 2: Intermediate genetic profile with low physical activity (n = 140) | Cluster 3: High-$MVS_{FTO}$ profile with moderate physical activity (n = 273) | p-value | Corrected p-value | | |
|---|---|---|---|---|---|---|---|---|
| Continuous covariates | | | Mean (95% CI) | | | 1 vs. 2 | 2 vs. 3 | 1 vs. 3 |
| Cluster-forming indicators | Multi-variant score ($MVS_{FTO}$) | 2.18 (2.13 – 2.23) | 3.01 (2.92 – 3.09) | 3.47 (3.40 – 3.54) | <0.001* | <0.001* | <0.001* | <0.001* |
| | Body Mass Index (kg/m²) | 25.91 (25.30–26.52) | 34.28 (33.47–35.09) | 24.84 (24.29–25.39) | <0.001* | <0.001* | <0.001* | 0.052 |
| | Leisure-Time Activity (MET-min/week) | 1,738.50 (1,470.54–2,006.46) | 339.77 (244.23–435.31) | 1,075.26 (888.07–1,262.45) | <0.001* | <0.001* | <0.001* | 0.014* |
| Categorical covariates | | | Prevalence in % (95%CI) | | p-value | Corrected p-value | | |
| Roma | | 49.39 (43.20–55.60) | 58.99 (50.70–66.91) | 47.25 (41.39–53.18) | 0.076 | 0.209 | 1.000 | 0.073 |
| Women | | 65.18 (59.09–70.92) | 65.47 (57.31–72.99) | 66.30 (60.54–71.71) | 0.963 | 1.000 | 1.000 | 1.000 |
| Education | Less than primary or primary | 53.04 (46.81–59.19) | 59.71 (51.43–67.60) | 51.28 (45.37–57.17) | 0.442 | 0.616 | 0.313 | 1.000 |
| | Vocational or high school | 38.06 (32.17–44.22) | 33.09 (25.68–41.20) | 37.36 (31.78–43.21) | | 0.991 | 1.000 | 1.000 |
| | College or university | 8.91 (5.83–12.94) | 7.19 (3.76–12.39) | 11.36 (8.00–15.52) | | 1.000 | 0.547 | 1.000 |
| Continuous covariates | | | Mean (95% CI) | | p-value | Corrected p-value | | |
| Age (years) | | 42.35 (40.76–43.93) | 46.40 (44.36–48.44) | 42.29 (40.85–43.73) | 0.003* | 0.007* | 0.004* | 1.000 |
| Waist-to-Height Ratio | | 0.56 (0.55–0.57) | 0.68 (0.67–0.69) | 0.54 (0.53–0.55) | <0.001* | <0.001* | <0.001* | 0.021* |
| Systolic BP (mmHg) | | 124.51 (122.51–126.51) | 131.61 (129.02–134.21) | 122.68 (120.80–124.56) | <0.001* | <0.001* | <0.001* | 0.555 |
| Diastolic BP (mmHg) | | 78.86 (77.70–80.02) | 82.72 (81.29–84.14) | 77.79 (76.67–78.91) | <0.001* | <0.001* | <0.001* | 0.444 |
| Domestic Physical Activity (MET-min/week) | | 3,007.17 (2,681.92–3,332.41) | 2,541.60 (2,068.14–3,015.07) | 3,160.73 (2,785.38–3,536.08) | 0.097 | 1.000 | 0.119 | 0.196 |
| GHQ-12 Likert Score | | 10.51 (9.97–11.05) | 10.61 (9.90–11.32) | 11.03 (10.37–11.70) | 0.956 | 1.000 | 1.000 | 1.000 |

Values are presented as mean (95% confidence interval) for continuous variables and percentages (95% confidence interval) for categorical variables. Omnibus p-values were evaluated using Kruskal–Wallis tests for continuous traits and chi-square tests for categorical distributions. Pairwise post-hoc comparisons were adjusted using the Bonferroni procedure. *: statistically significant after multiple comparison adjustment (omnibus $p < 0.05$, and post-hoc adjusted $p < 0.05$).
