# Supplementary material for: Associations Between FTO (Fat Mass- and Obesity-Associated) Gene Allelic Load and Heart Rate Responses to Submaximal Exercise in Humans: A Cross-Sectional Study
Source: Biology (Basel). 2026 Sep 11;15(18):1607. doi: 10.3390/biology15181607 (PMC13604555; doi:10.3390/biology15181607)
Supplement: Supplementary file 1 [file biology-15-01607-s001.zip › Supplementary Table S7.pdf]

Supplementary Table S7. Complete structural equation model (SEM) path coefficients and parameter estimates for post-exercise heart rate traits

Model A	Independent Predictor (Path)	Unstandardized Coef. (β)	Robust Std. Err. (SE)	z-statistic	95% Confidence Interval	p-value
BMI	MVS_{FTO}	0.839	0.305	2.75	(0.241 – 1.437)	0.006*
LTPA	MVS_{FTO}	188.525	84.771	2.22	(22.377 – 354.673)	0.026*
LTPA	BMI	-2.266	10.812	-0.21	(-23.457 – 18.925)	0.834
HR_{aft}	MVS_{FTO} (Direct path)	-4.576	1.348	-3.39	(-7.218 – -1.934)	0.001*
HR_{aft}	LTPA	-0.003	0.0006	-5.12	(-0.004 – -0.002)	< 0.001*
HR_{aft}	BMI	0.204	0.167	1.22	(-0.123 – 0.531)	0.221
**Model B**	**Independent Predictor (Path)**	**Unstandardized Coef. (β)**	**Robust Std. Err. (SE)**	**z-statistic**	**95% Confidence Interval**	**p-value**
BMI	MVS_{FTO}	0.836	0.305	2.74	(0.238 – 1.434)	0.006*
LTPA	MVS_{FTO}	187.824	84.770	2.22	(21.678 – 353.970)	0.027*
LTPA	BMI	-2.664	10.801	-0.25	(-23.834 – 18.506)	0.805
ΔHR	MVS_{FTO} (Direct path)	-4.036	1.223	-3.30	(-6.433 – -1.639)	0.001*
ΔHR	LTPA	-0.002	0.0005	-4.38	(-0.003 – -0.001)	< 0.001*
ΔHR	BMI	-0.024	0.158	-0.15	(-0.334 – 0.286)	0.879
**Model C**	**Independent Predictor (Path)**	**Unstandardized Coef. (β)**	**Robust Std. Err. (SE)**	**z-statistic**	**95% Confidence Interval**	**p-value**
BMI	MVS_{FTO}	0.839	0.305	2.75	(0.241 – 1.437)	0.006*
LTPA	MVS_{FTO}	187.788	84.770	2.22	(21.642 – 353.934)	0.027*
LTPA	BMI	-2.104	10.801	-0.19	(-23.274 – 19.066)	0.845
$HR_{max\%}$	MVS_{FTO} (Direct path)	-3.033	0.774	-3.92	(-4.550 – -1.516)	< 0.001*
$HR_{max\%}$	LTPA	-0.002	0.0003	-5.87	(-0.003 – -0.001)	< 0.001*
$HR_{max\%}$	BMI	0.284	0.099	2.87	(0.090 – 0.478)	0.004*

Estimates were obtained via maximum likelihood structural equation modeling in Stata v17.0 corresponding to the path diagrams presented in Figure 4. Models evaluate the simultaneous direct and indirect pathways linking the multi-variant score for *FTO* (MVS_{FTO}), Body Mass Index (BMI, kg/m²), and Leisure-Time Physical Activity (LTPA, MET-min/week) to immediate post-exercise heart rate (HR_{aft}, Panel A), delta heart rate response (ΔHR, Panel B), and percentage of age-predicted maximal heart rate ($HR_{max\%}$, Panel C). All three structural path models are fully saturated (degrees of freedom = 0), serving strictly as multivariate path-analytic parameter estimation tools across the observed system rather than confirmatory goodness-of-fit models. Model covariates and baseline clinical adjustments are identical to those applied in fully adjusted regression specifications. Unstandardized regression coefficients (Coef. [β]) represent the direct or indirect structural path estimates, and Robust Std. Err. (SE) denotes robust standard errors. The z-statistic reflects the Wald test statistic for parameter significance, alongside corresponding 95% Confidence Intervals and exact p-values. *: statistically significant structural path under the exploratory path-analytic framework ($p < 0.05$).
